# Role of WNT5A receptors FZD5 and RYK in prostate cancer cells

**DOI:** 10.18632/oncotarget.25551

**Published:** 2018-06-05

**Authors:** Stefanie Thiele, Ariane Zimmer, Andy Göbel, Tilman D. Rachner, Sandra Rother, Susanne Fuessel, Michael Froehner, Manfred P. Wirth, Michael H. Muders, Gustavo B. Baretton, Franz Jakob, Martina Rauner, Lorenz C. Hofbauer

**Affiliations:** ^1^ Division of Endocrinology and Metabolic Bone Diseases, Department of Medicine III, Dresden, Germany; ^2^ Center for Healthy Aging, Technische Universität Dresden Medical Center, Dresden, Germany; ^3^ Institute of Materials Science, Max Bergmann Center of Biomaterials, Technische Universität Dresden, Dresden, Germany; ^4^ Department of Urology, Technische Universität Dresden, Dresden, Germany; ^5^ Institute of Pathology, Technische Universität Dresden, Dresden, Germany; ^6^ Orthopedic Center for Musculoskeletal Research, University of Würzburg, Würzburg, Germany; ^7^ German Cancer Consortium (DKTK), partner site Dresden and German Cancer Research Center (DKFZ), Heidelberg, Germany

**Keywords:** prostate cancer, WNT5A, Wnt receptors, Frizzled, RYK

## Abstract

Prostate cancer is the most common malignancy in men and has a high propensity to metastasize to bone. WNT5A has recently been implicated in the progression of prostate cancer, however, the receptors that mediate its effects remain unknown. Here, we identified Wnt receptors that are highly expressed in prostate cancer and investigated which of these receptors mediate the anti-tumor effects of WNT5A in prostate cancer *in vitro*.

Extensive *in vitro* analyses revealed that the WNT5A receptors FZD5 and RYK mediate the anti-tumor effects of WNT5A on prostate cancer cells. Knock-down of FZD5 completely abrogated the anti-proliferative effect of WNT5A in PC3 cells. In contrast, knock-down of RYK and FZD8 did not rescue the inhibition of proliferation after WNT5A overexpression. In contrast, RYK knock-down inhibited the pro-apoptotic effect of WNT5A in PC3 cells by 60%, whereas the knock-down of either FZD5 or FZD8 further stimulated apoptosis after WNT5A overexpression (by 33% and 234%, respectively). Surface plasmon resonance analysis indicated that WNT5A has a 30% stronger binding response to FZD5 than to RYK. Further investigations using a tissue microarray revealed that expression of RYK is increased in advanced prostate cancer tumor stages, but is not associated with survival of prostate cancer patients. In contrast, patients with low local FZD5 expression, in particular in combination with low WNT5A expression, showed a longer disease-specific survival.

In conclusion, WNT5A/FZD5 and WNT5A/RYK signaling are both involved in mediating the pro-apoptotic and anti-proliferative effects of WNT5A in prostate cancer.

## INTRODUCTION

Prostate cancer is the most common malignancy and the third most frequent cause of death from cancer in older men. The occurrence of metastases in more than 80% of the patients with advanced prostate cancer is the main reason for the high morbidity and mortality of the disease [1]. A characteristic hallmark of metastatic prostate cancer is the formation of osteosclerotic lesions which form bone of poor quality that is prone to pathological fractures [2]. Despite the high incidence of bone metastases, the metastatic process as well as the involved tumor and bone marrow-derived factors largely remain elusive.

Recent studies show that molecules of the Wnt family play a decisive role in the development of bone metastases [3]. A key ligand of this protein family is the Wingless-type MMTV integration site family member 5A (WNT5A), which has an important regulatory role on different cell functions and also in many cancers [4]. In prostate cancer, WNT5A exerts potent anti-tumor effects by suppressing tumor cell proliferation and inducing apoptosis [5]. While most Wnt ligands activate either canonical or non-canonical Wnt signaling, WNT5A was shown to interact with both signaling pathways [6]. Signal transduction of WNT5A and other Wnt ligands further depends on the tissue-specific availability of Wnt receptors [6]. Two main classes of Wnt receptors can be distinguished: the Frizzled (FZD) receptors and the receptor tyrosine kinases (RTKs). Besides those, low-density lipoprotein (LDL) receptor related proteins (LRP) 5 and 6 co-receptors also exist [7]. The FZD receptors were implicated as Wnt receptors much earlier than the RTKs related to receptor-like tyrosine kinase (RYK) and receptor tyrosine kinase-like orphan receptor (ROR) [8, 9]. Structurally, FZD receptors fundamentally differ from RTKs. FZD receptors are composed of a seven-transmembrane domain, adjunctive to an extracellular cysteine-rich domain binding multiple Wnt molecules. ROR1 and ROR2 represent single transmembrane receptor tyrosine kinases, comprising a cysteine rich domain comparable to FZD receptors [10]. As co-receptors they form complexes with FZD receptors, but can also act as independent receptors, directly transmitting signals after ligand binding. Similarly, RYK - an atypical RTK lacking detectable catalytic activity [11] - is a single transmembrane receptor that is mostly known for its homeostatic role in the central nervous system [12–16]. Recent studies identified WNT5A to interact with various members of the FZD family (FZD2, 3, 4, 7, 8) as well as the co-receptors ROR2 and RYK [7]. Moreover, WNT5A and its receptors have been implicated in different human cancer entities including glioblastoma, leukemia, and pancreatic cancer [17–23]. After having previously shown that WNT5A exerts potent anti-tumor effects in prostate cancer cells [5], this study aimed to investigate which of the receptors mediates its anti-tumor effects.

## RESULTS

### FZD5 and RYK distinctly mediate WNT5A effects on prostate cancer cell proliferation and apoptosis

**Figure 1:**
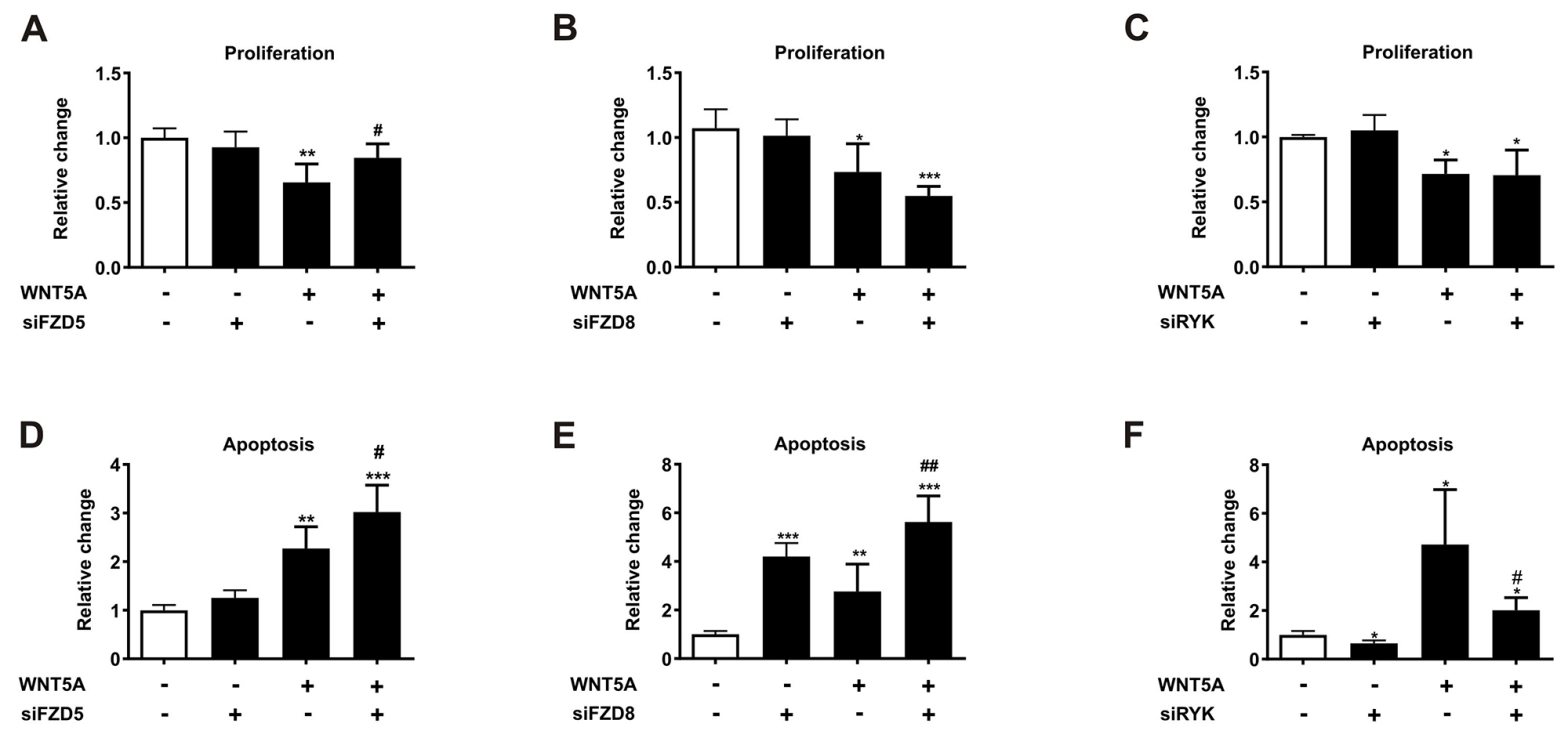
FZD5 and RYK mediate the anti-proliferative and pro-apoptotic effects of WNT5A in PC3 cells Cells were either single treated using siRNAs (siFZD5, siFZD8, siRYK) or WNT5A plasmid-DNA (pcDNA3.1WNT5A), or treated with a combination of first siRNAs (24 h) and afterwards WNT5A plasmid-DNA (24 h). **(A-C)** Proliferation of control (white bars) and treated (black) PC3 cells. Proliferation was measured using a BrdU proliferation assay after 48 h. **(D-F)** Caspase 3/7 activation of control (white bars) and treated (black) PC3 cells. Apoptosis was measured after 48 h with a Caspase Glo^®^ 3/7 assay. Data are shown as mean ± SD. ^*^p<0.05; ^**^p<0.01, ^***^p<0.001; ^#^p<0.05; ^##^p<0.01. ^*^ vs. control, ^#^ vs. WNT5A overexpression.

After demonstrating that WNT5A reduces prostate cancer cell proliferation and induces apoptosis [5], we sought to investigate which receptors were responsible for these effects. Thus, we first analyzed the expression pattern of seven known WNT5A receptors in two human (PC3, C4-2B) and two murine (RM1, TRAMPC2) prostate cancer cell lines. FZD7, FZD8, and ROR2 showed the lowest expression in most of the prostate cancer cell lines, while FZD6 and RYK were abundantly expressed (Supplementary Figure 1). To determine which receptors mediate the anti-proliferative and pro-apoptotic effects of WNT5A in prostate cancer, we knocked-down each of the receptors with specific siRNAs in PC3 cells 24 h before the induction of WNT5A overexpression. After validating the successful knock-down and overexpression using qPCR (Supplementary Figure 2) we assessed proliferation by determining BrdU integration and apoptosis by caspase 3 and 7 activation. The knock-down of FZD5 completely reversed the suppressive effect of WNT5A on proliferation (Figure 1A). However, FZD8 and RYK knock-down failed to prevent the suppression of proliferation by WNT5A (Figure 1B, 1C). Interestingly, the increased rate of apoptosis after WNT5A overexpression was not reversed by FZD5 or FZD8 knock-down, but even further increased (Figure 1D, 1E). However, knock-down of RYK nearly fully reversed the induction of apoptosis after WNT5A overexpression (Figure 1F). Besides its effect on WNT5A induced apoptosis, RYK also slightly reduces apoptosis in control cells suggesting a role independent from WNT5A signaling. Knock-down of FZD2, FZD6, FZD7, and ROR2 receptors did not mediate the effects of WNT5A neither on proliferation nor on apoptosis (Supplementary Figure 3). These findings suggest that FZD5 and RYK mediate distinct anti-tumor effects of WNT5A on prostate cancer cells.

### WNT5A binds FZD5 and RYK

**Figure 2:**
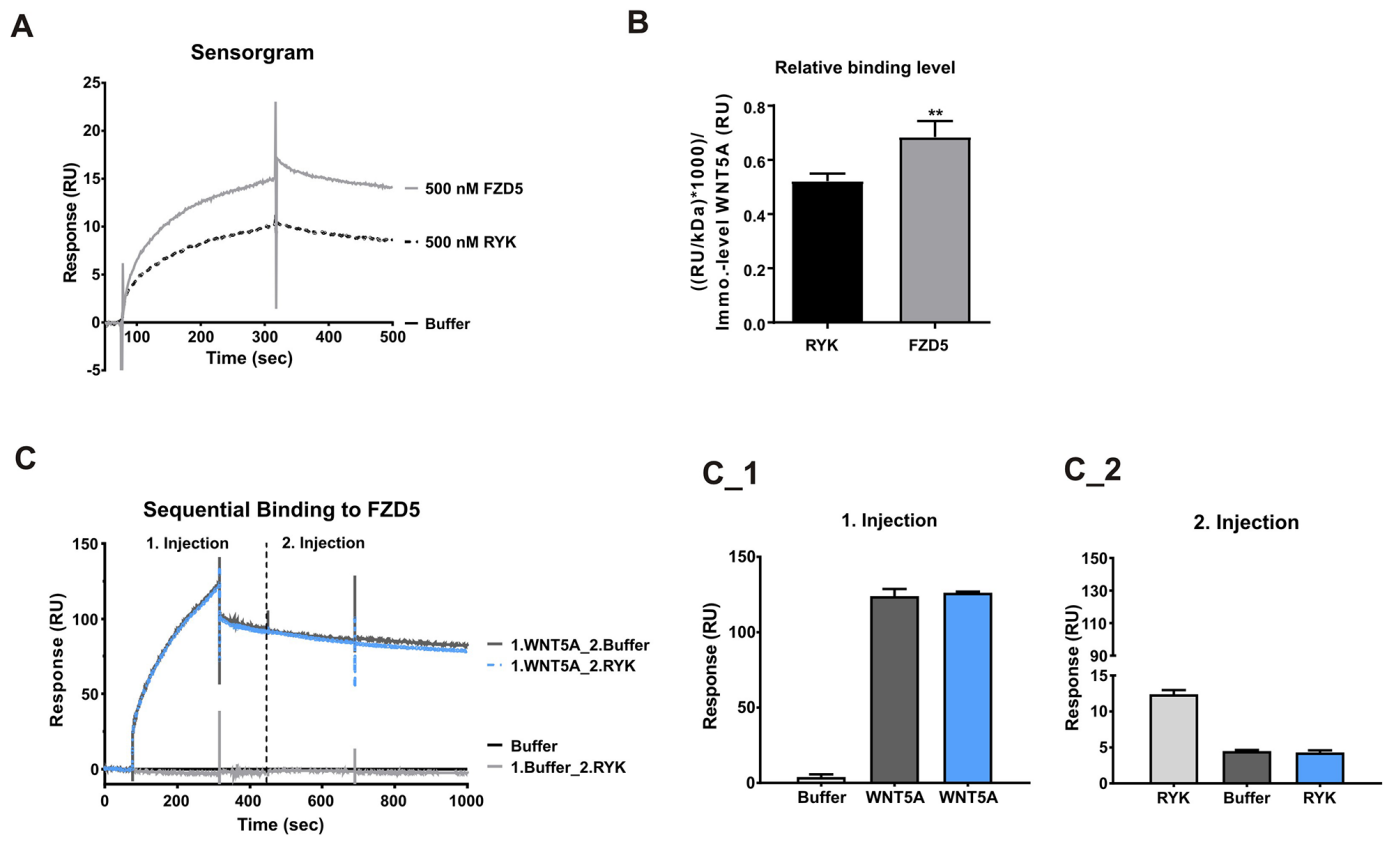
WNT5A showed higher binding response to FZD5 **(A-B)** Binding response of FZD5 (500 nM) and RYK (500 nM) to immobilized WNT5A. The interaction was displayed in a representative sensorgram. **(C-C_2)**, Sequential binding of WNT5A (100 nM) and/or RYK (200 nM) to immobilized FZD5.

To assess if WNT5A has a preferred receptor, surface plasmon resonance analysis was performed. Although high concentrations were required, interactions of WNT5A with both Wnt receptors RYK and FZD5 were detected. However, the binding response of FZD5 to WNT5A was 31% higher than the binding to RYK (Figure 2A–2B). Because RYK is described to act as single receptor but also as co-receptor for different FZD receptors [15], we also immobilized FZD5 on the sensor chip and examined if RYK can directly bind FZD5. After first just injecting buffer as negative control, RYK was added at the second injection. Sensogram evaluation and quantification showed a marginal binding response of RYK to FZD5 (Figure 2C–2C_2). Furthermore, sequential binding was used to analyze if WNT5A has a second receptor binding site for RYK, when already bound to FZD5. Therefore, we first injected WNT5A, to see the expected binding response to FZD5 (Figure 2C–2C_1) and then injected RYK or buffer as control. Neither the buffer nor RYK injection led to a binding response after the WNT5A injection (Figure 2C, 2C_2). From these data we conclude, that RYK does not act as a co-receptor for FZD5 and that WNT5A can bind either FZD5 or RYK at the same binding site.

### FZD5 and RYK, but not ROR2 mRNA expression correlate with the prostate cancer tumor stage

**Figure 3:**
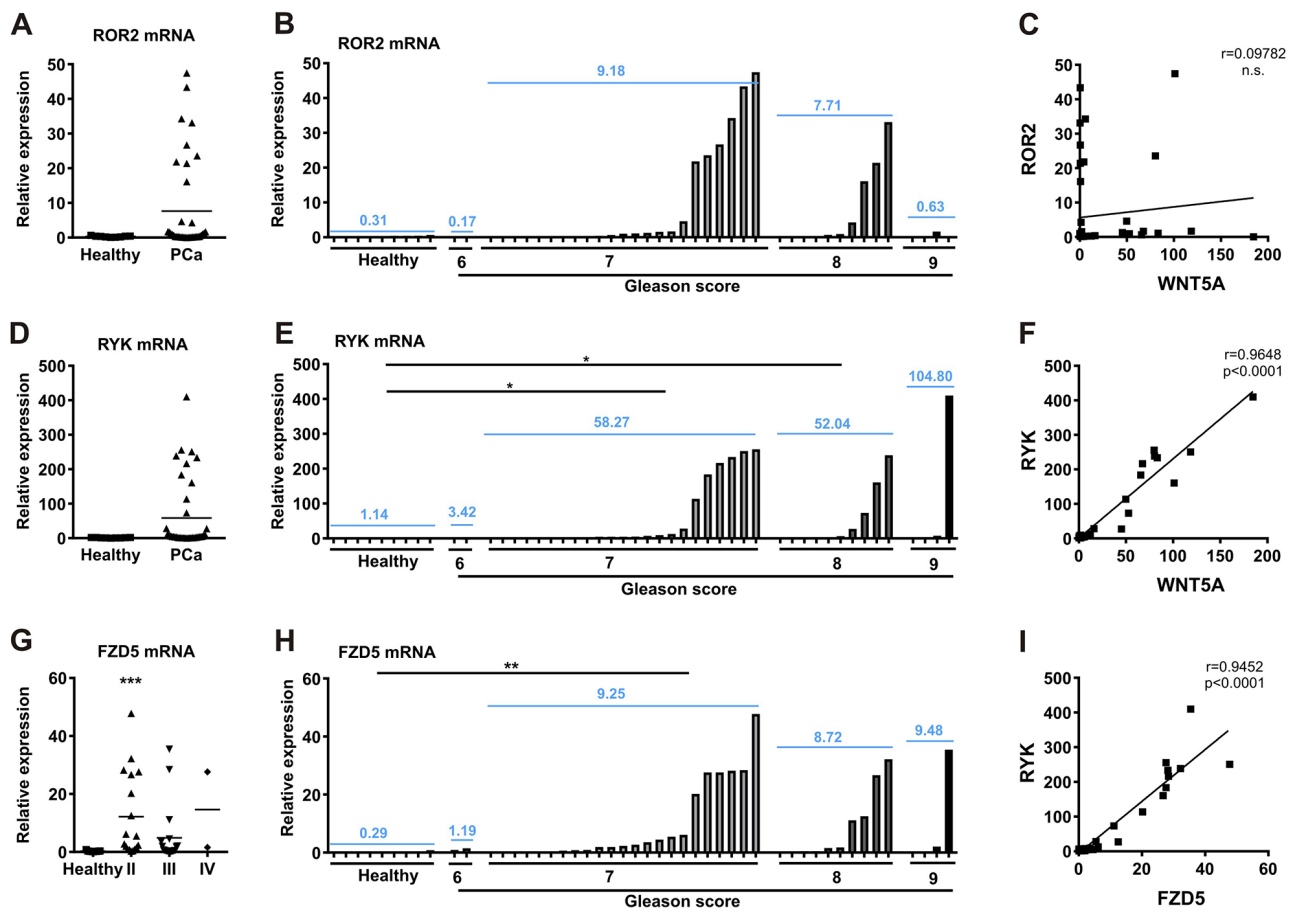
RYK and FZD5 are increased in prostate cancer and show correlating expression patterns (A) cDNA prostate cancer array was used to measure **(A-B)**, ROR2; **(D-E)**, RYK; and **(G-H)**, FZD5 expression in different tumor stages of prostate cancer. Tumor stages are stated according to tumor growth (stage II-IV) and degree of differentiation (GS 6-9). **(C)** No correlation of ROR2 and WNT5A expression in prostate cancer cDNA samples. **(F-I)** Positive correlation of RYK with WNT5A and FZD5. Expression was analyzed by qPCR and normalized to β-actin. Data are shown as mean (blue). Correlation was calculated using Pearson r correlation analysis and linear regression. ^*^p<0.05; ^**^p<0.01; ^***^p<0.001 vs. healthy controls.

To analyze the expression of the receptors in prostate cancer, a cDNA prostate cancer array, including nine healthy and 39 tumor samples was assessed for expression of ROR2, RYK, and FZD5. The characteristics of the cohort are described in Supplementary Table 2. ROR2 mRNA expression showed no differences between healthy and tumor samples and was also not significantly increased in patients categorized by GS (Figure 3A, 3B). In contrast to RYK and FZD5, ROR2 exhibited no correlation with WNT5A expression (Figure 3C, 3F; [5]). The mRNA expression level of FZD5 was significantly higher in stage II prostate cancer patients compared to healthy controls and higher prostate cancer stages (p<0.01; p<0.001, respectively) (Figure 3D, 3G). RYK expression showed no significant differences between healthy and tumor samples. However, RYK levels were increased in patients with GS 7 and 8 and FZD5 expression in GS 7 compared to healthy controls (Figure 3E, 3H). There was no correlation between the expression patterns of ROR2 and RYK or FZD5 (data not shown), but RYK and FZD5 expression were positively correlated (Figure 3I, r=0.9452, p<0.001). These results underline a role of the Wnt receptors FZD5 and RYK as transducers of WNT5A action in prostate cancer.

### Local FZD5 expression as a potential disease-specific survival marker in prostate cancer

To assess the clinical significance of WNT5A receptors in human prostate cancer, FZD5 and RYK protein levels were analyzed in a large patient cohort with different tumor stages. The clinical characteristics of the patient cohort are summarized in Supplementary Table 2. While FZD5 immunohistochemistry showed a uniform cytoplasmic and membranous staining, RYK showed a heterogeneous staining pattern. Several patients showed a cytoplasmic staining of RYK close to the cell membrane, while a predominantly nuclear staining was found in others. Most tissue cores of the prostate cancer and tumor-free and BPH tissues exhibited a mixture of cytoplasmic and nuclear staining (Supplementary Figure 4). As cleavage of the RYK receptor by γ-secretases has previously been reported in neurogenesis [24], evaluation of RYK staining was classified into cytoplasmic RYK (RYK) and nuclear RYK (RYK ICD) receptor expression.

For survival analysis, patients were divided into two groups at the median of the receptor expression. Overall and disease-specific survival was also not different in groups with low vs. high expression of FZD5, RYK, and RYK-ICD, respectively (Table 1). However, high FZD5 expression tended to be associated with a longer disease-specific survival (Median time: low: 10.8 years; high: 11.5 years, p=0.079). In addition, we found a significantly longer disease-specific survival in patients with low WNT5A and low FZD5 expression (median time: 11.9 years), compared to the patients with high WNT5A and high FZD5 expression (median time: 9.2 years, p=0.049). While 15 years after radical prostatectomy, six patients were at risk of death in the “WNT5A/FZD5 high” group, only one patient was at risk in the “WNT5A/FZD5 low” expression group. Also patients with the combination of low FZD5/high RYK expression exhibited a tendency for a longer disease-specific survival than patients with high FZD5/low RYK expression (p=0.075, Table 1). These findings indicate that local FZD5 expression, in particular in combination with WNT5A, may be a prognostic disease-specific survival marker for prostate cancer.

**Table 1:** Univariate Cox proportional-hazards regression analysis of factors influencing overall and disease specific survival

	Groups	n (%)	Overall survival		Disease survival	
			HR (95% CI)	P-value	HR (95% CI)	P-value
FZD5	High	200 (50)	0.889 (0.95-3.39)	0.534	1.795 (0.95-3.39)	0.079
	Low	200 (50)	1.124 (0.30-1.05)		0.557 (0.30-1.05)	
RYK	High	200 (50)	0.957 (0.66-1.39)	0.818	0.877 (0.46-1.66)	0.685
	Low	200 (50)	1.045 (0.72-1.52)		1.140 (0.60-2.15)	
RYK ICD	High	200 (50)	1.159 (0.80-1.68)	0.434	0.774 (0.41-1.46)	0.434
	Low	200 (50)	0.863 (0.59-1.25)		1.291 (0.68-2.44)	
WNT5A and FZD5	WNT5A High FZD5 High	114 (28.5)	0.953 (0.59-1.55)	0.845	2.430 (1.05-5.61)	**0.049**
	WNT5A Low FZD5 Low	114 (28.5)	1.049 (0.65-1.71)		0.411 (0.18-0.95)	
	WNT5A High FZD5 Low	86 (21.5)	1.088 (0.61-1.95)	0.775	0.504 (0.18-1.39)	0.202
	WNT5A Low FZD5 High	85 (21.25)	0.919 (0.51-1.65)		1.984 (0.72-5.46)	
WNT5A and RYK	WNT5A High RYK High	111 (27.75)	1.044 (0.62-1.75)	0.872	1.301 (0.58-2.95)	0.530
	WNT5A Low RYK Low	114 (28.5)	0.958 (0.57-1.61)		0.769 (0.34-1.74)	
	WNT5A High RYK Low	111 (27.75)	1.355 (0.80-2.29)	0.247	1.190 (0-49-2-87)	0.695
	WNT5A Low RYK High	89 (22.25)	0.738 (0.44-1.25)		0.840 (0.35-2.03)	
WNT5A and RYK ICD	WNT5A High RYK ICD High	96 (24)	1.191 (0.69-2.07)	0.529	1.142 (0.45-2.88)	0.776
	WNT5A Low RYK ICD Low	96 (24)	0.840 (0.48-1.46)		0.876 (0.35-2.21)	
	WNT5A High RYK ICD Low	104 (26)	0.896 (0.54-1.49)	0.671	1.759 (0.73-4.23)	0.221
	WNT5A Low RYK ICD High	104 (26)	1.116 (0.67-1.85)		0.569 (0.24-1.37)	
FZD5 and RYK	FZD5 High RYK High	133 (33.25)	0.887 (0.55-1.43)	0.620	1.742 (0.73-4.19)	0.225
	FZD5 Low RYK Low	133 (33.25)	1.127 (0.70-1.81)		0.574 (0.24-1.38)	
	FZD5 High RYK Low	67 (16.75)	0.979 (0.54-1.78)	0.945	2.458 (0.98-6.20)	0.075
	FZD5 Low RYK High	67 (16.75)	1.021 (0.56-1.86)		0.407 (0.16-1.03)	
FZD5 and RYK ICD	FZD5 High RYK ICD High	86 (21.5)	1.125 (0.61-2.08)	0.705	2.091 (0.67-6.49)	0.217
	FZD5 Low RYK ICD Low	86 (21.5)	0.889 (0.48-1.64)		0.478 (0.15-1.48)	
	FZD5 High RYK ICD Low	114 (28.5)	0.798 (0.78-2.01)	0.338	1.973 (0.91-4.26)	0.099
	FZD5 Low RYK ICD High	116 (29)	1.253 (0.50-1.28)		0.507 (0.24-1.10)	

Abbreviations: CI, Confidence interval; FZD, Frizzled; HR, Hazard ratio; ICD, Intracellular domain; n, frequency; RYK, Related to receptor tyrosine kinase.

### Expression of FZD5 and RYK receptors in prostate cancer tissue

**Figure 4:**
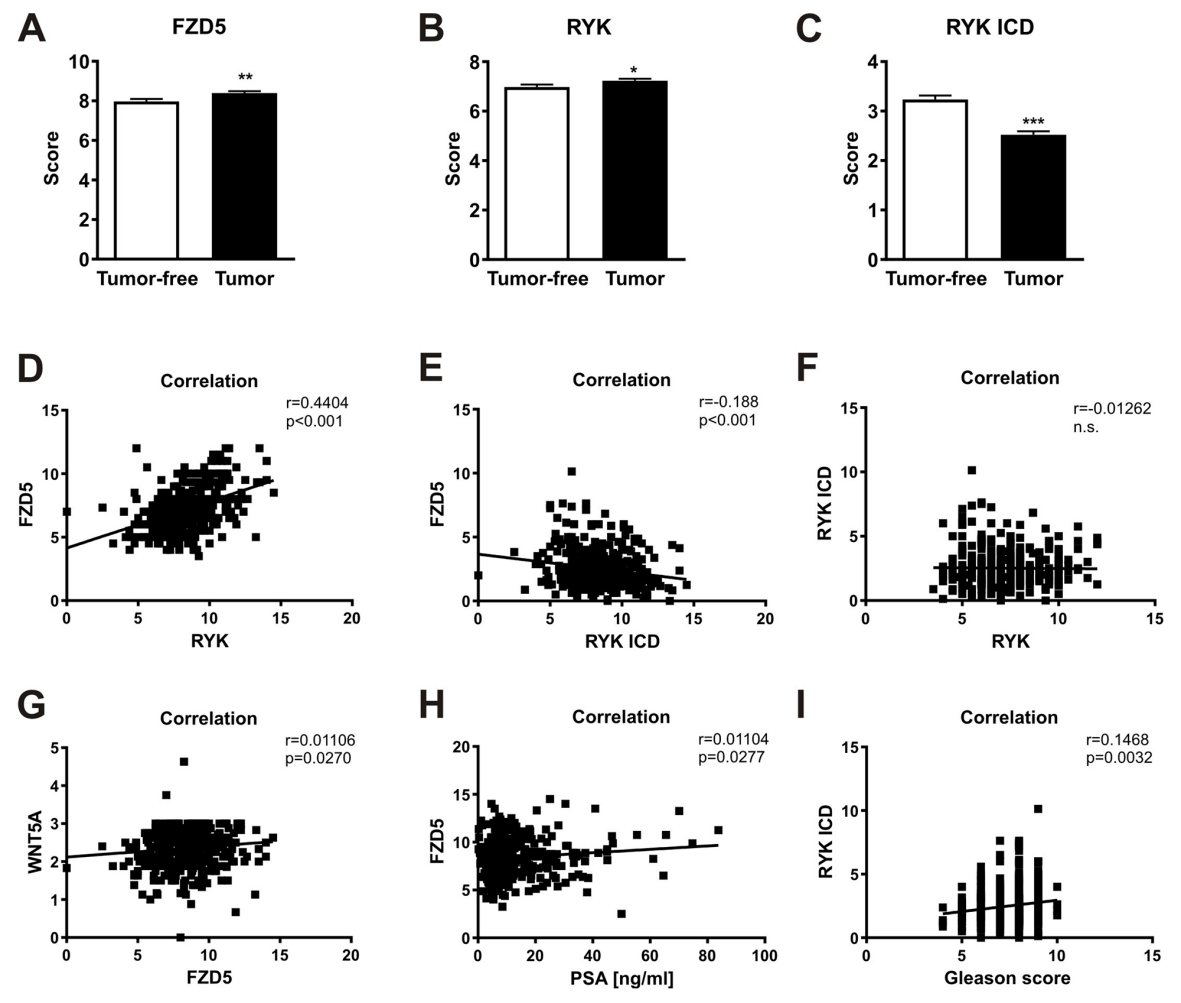
Wnt receptors as potential prognostic markers in prostate cancer **(A-C)** FZD5, RYK, and RYK-ICD expression in tumor and adjacent tumor-free cores of the TMA. **(D-I)**, Correlation analyses of receptors with each other and different clinical factors. Data of TMA are shown as mean ± SD. Correlation was calculated using Pearson r correlation analysis and linear regression. ^*^p<0.05; ^**^p<0.01; ^***^p<0.001.

In line with the cDNA array, the TMA also revealed that FZD5 and RYK expression levels were significantly higher in malignant tissues compared to adjacent tumor-free tissues of the same patients and also compared to BPH patients (Figure 4A–4B, data not shown). In contrast to this, RYK ICD staining, was significantly lower in tumor compared to tumor-free tissue (Figure 4C, p<0.001) and RYK ICD expression was also lower in tumor tissue vs. BPH patients (data not shown). Analysis of the staining patterns of the receptors in tumor tissue showed a positive correlation between FZD5 and RYK expression (Figure 4D, r=0.4404, p<0.001). RYK and RYK-ICD staining, however, did not correlate with each other or with WNT5A (Figure 4F, data not shown, respectively). FZD5 and RYK-ICD (Figure 4E, r=-0.188, p<0.001) and FZD5 and WNT5A (Figure 4G, r=0.01106, p=0.027) expression yielded significant results after correlation analysis. Further correlation analysis with clinicopathological markers for prostate cancer revealed a significant correlation of FZD5 expression and the PSA level (Figure 4H, r=0.01104, p=0.028) and between RYK-ICD and GS (Figure 4I, r=0.1468, p=0.003). Although the positive correlation of FZD5 protein with WNT5A protein expression in the prostate cancer samples is in line with the cDNA array the p-values in Figure 4E, 4G, 4H, and 4I indicate non-correlations deriving from the large number of 400 patients included in the TMA.

These findings confirm the predominant role of FZD5 as a receptor for WNT5A, but also highlight a potential relevance of the intracellular part of RYK.

## DISCUSSION

To date, the role of WNT5A is controversially disputed [25] as it has been attributed to tumor-suppressive functions [26–29] but also associated with negative outcomes. In prostate cancer we showed that WNT5A exerts anti-tumor effects [5]. By analyzing the expression of known WNT5A receptors in five different prostate cancer cell lines, performing functional assays, and validating receptor expression in a large set of human samples, we found the receptors FZD5 and RYK mediate anti-tumor effects of WNT5A in prostate cancer.

Deregulated WNT5A/ROR2 signaling appears to be of great importance in cancer. While WNT5A/ROR2 signaling acts tumor suppressive in hepatocellular carcinoma [30], gastric carcinoma [31], and myelogenous leukemia [32], it enhances proliferation of chronic lymphocytic leukemia [21]. Because of its critical role in different cancer entities, we expected a striking effect of ROR2 in prostate cancer. However, ROR2 was barely expressed in prostate cancer cell lines and hence, also the knock-down did not influence proliferation and apoptosis. To assess if this was limited to our *in vitro* studies, we analyzed ROR2 mRNA expression in a prostate cancer cDNA array. Similarly, ROR2 was not differentially expressed in healthy and primary prostate cancer tissue and did not correlate with WNT5A expression. In contrast to our findings of ROR2 in primary tumors, Tseng et al. reported a reduced mRNA expression of ROR2 in metastatic prostate tumors [33]. Furthermore prostate cancer invasion has been described to depend on ROR2 and FZD2 [34]. Therefore, ROR2 may not mediate the anti-tumor effects of WNT5A in primary prostate cancer, but may have a more significant role in metastasized prostate cancer stages.

The phenotype of RYK-deficient mice [35] resembles that of WNT5A [36] and ROR2 [37] deficient mice. Therefore, it is likely that similar pathways are regulated. WNT/RYK signaling is described in numerous cancers including glioblastoma, melanoma, and breast cancer [18, 19, 19–38, 39]. In prostate cancer, RYK has been shown to be expressed in primary prostatic tissue [40] and to be upregulated in DU-145 prostate cancer cells after antiestrogen treatment [41]. Therefore, we hypothesized that RYK may also act tumor diminishing in prostate cancer. After demonstrating *in vitro* that RYK knock-down reverses the effect of WNT5A on apoptosis and has no effect on proliferation, we investigated its expression in primary prostate cancer tissue. This study revealed that mRNA and protein expression of RYK is increased in advanced prostate cancer tumor stages. However, its expression was not associated with the survival of prostate cancer patients. Interestingly, RYK ICD (nuclear) showed an opposite expression pattern. This underlines the importance to distinguish between the cleavage products of RYK. As RYK is described to transduce Wnt signals either directly or as co-receptor bound to FZD-receptors [15] and because of the strong correlation between RYK and FZD5 in the tissue analyses of the patients, we also investigated if RYK can bind to FZD5 using surface plasmon resonance. However, this was negligible compared to the WNT5A/FZD5 interplay, suggesting that RYK may not function as a FZD5 co-receptor.

Amongst all FZD receptors, FZD5 was one of the most highly expressed receptors in the prostate cancer cell lines and WNT5A also showed a stronger binding response to FZD5 than to RYK. Silencing of FZD5 diminishes prostate cancer burden *in vivo* [42], which was confirmed by our results *in vitro*. Interestingly, while the knock-down of FZD5 alone had no effect on cell proliferation or apoptosis, co-treatment with WNT5A further enhanced WNT5A-induced apoptosis, while it normalized cell proliferation. The reason why the increase in apoptosis was not reflected by a decreased proliferation rate is unclear, but may indicate that these processes are uncoupled and may depend on different receptor constellations. Moreover, we have previously shown that FZD5 mRNA expression is upregulated in human prostate cancer and correlates with WNT5A expression [5, 43]. Our study now extends these data identifying high FZD5 protein levels in prostate cancer tissue as well. Another study linked FZD5 to the aggressiveness of prostate cancer [33]. In our patient cohort we were not able to confirm this for FZD5 alone, but the combined analysis of WNT5A and FZD5 expression showed that patients with low WNT5A and low FZD5 expression had a longer disease-specific survival than patients with high WNT5A and high FZD5 expression. Besides prostate cancer FZD5 is downregulated in endometrial adenocarcinoma [44]. In addition, motility and invasion of melanoma cells is increased through WNT5A/FZD5 signaling [45]. Taken together, the data suggest a more prominent role of WNT5A/FZD5 than WNT5A/RYK signaling in prostate cancer.

Despite the novel findings of WNT5A receptors in prostate cancer, our study has potential limitations. These include the mortality data of the TMA cohort. Although it comprised 400 patients, only 34 patients died of the disease within the median follow-up period of 8.8 years. This reflects the effective early detection of prostate cancer at a curable stage. As WNT5A can activate both, canonical and non-canonical Wnt signaling, several downstream pathways can be affected by influencing receptor expression. Thus, analyzing the downstream mechanisms after a specific receptor knock-down will be critical to determine which pathways act on which cellular functions.

In conclusion, this study suggests that FZD5 and RYK are the predominant receptors for mediating the pro-apoptotic and anti-proliferative effects of WNT5A in prostate cancer *in vitro*. Moreover, FZD5 and WNT5A co-expression exhibits potential as disease specific survival marker for prostate cancer patients.

## MATERIALS AND METHODS

### Ethics statement

The study was approved by the ethics committee of the Internal Review Board of the Technische Universität Dresden (EK194092004, EK195092004, and EK59032007).

### Tissue microarray

The patient cohort used in the tissue microarray (TMA) in the present study has been described previously [5]. Briefly, all patients underwent radical prostatectomy between 1996 and 2005 at the Department of Urology of the TU Dresden Medical Center, Dresden, Germany. None of the patients received neoadjuvant hormonal therapy. Patient characteristics are summarized in Supplementary Table 2. The TMA was constructed of mainly high-risk prostate cancer patients with Gleason score (GS)>7. The intermediate risk group included all patients with GS=7 and also the patients with GS<7 but pN1 (= regional lymph node metastasis acc. UICC). Low-risk patients had a GS<7 without metastasis. Fourteen paraffin blocks were constructed including four carcinoma and two adjacent tumor-free tissue cores of every patient. Clinical follow-up data with regard to time of death were available from all patients until 04/2016. Moreover, control samples from non-malignant tissue specimens of 41 patients with benign prostatic hyperplasia (BPH) were included.

### Immunohistochemistry and TMA scoring

Two μm thick TMA sections were stained for the WNT5A receptors FZD5 and RYK. Immunohistochemical procedure was performed as described previously [5]. FZD5 antibody (Abcam, Cambridge, UK) was used in a 1:100 dilution and RYK antibody (Abcam) 1:100. Scoring of the cores was done by two experienced scientists who were blinded to the clinical information. Discrepant findings were re-evaluated. Staining for FZD5 and RYK was assessed based on the staining intensity 0 (no staining), 1 (weak staining), 2 (moderate staining), or 3 (strong staining) and on the percentage of the stained tumor area 0 (0%), 1 (1-25%), 2 (26-50%), 3 (51-75%), or 4 (76-100%). Staining score including intensity and quantity ranging from 0 to 12 was calculated by multiplication. Nuclear staining was evaluated according to the same system. In the further course, mean values of staining of the malignant or benign cores were used.

### Cell culture

The human prostate cancer cell lines, PC3 (ATCC) and C4-2B (a kind gift from Prof. E. Keller, Ann Arbor, MI, USA) were cultured in RPMI 1640 medium (Thermo Fisher Scientific, Schwerte, Germany) containing 10% or 20% fetal bovine serum (FBS; Biochrome, Berlin, Germany) and 1% penicillin/streptomycin (P/S; Sigma-Aldrich, Hamburg, Germany), respectively. Murine prostate cancer cell lines RM1 and TRAMP-C2 (a kind gift from Prof. M. King, Cornell University, NY, USA) were cultivated in DMEM medium (Thermo Fisher Scientific) containing 10% FBS and 1% P/S. Cells were cultured at 37°C in humidified atmosphere (5% CO_2_). All cells were regularly authenticated and matched with short tandem repeat DNA profiles of the original cancer cell lines.

### Overexpression and knock-down experiments

For overexpression, 1 μg WNT5A plasmid-DNA (pcDNA3.1WNT5A, a kind gift from Qian Tao [46]) and 3 μl of the transfection reagent Fugene^®^ HD (Roche, Mannheim, Germany) was used. Plasmid-DNA was diluted in Opti-MEM^®^ (Thermo Fisher Scientific), and mixed with Fugene^®^ HD. After 20 min incubation at RT transfection mixture was added drop-wise to 70% confluent cells for 48 h. Knock-down of FZD2, FZD5, FZD6, FZD7, FZD8, ROR2, and RYK was performed using DharmaFECT1 (Thermo Fisher Scientific) and specific siRNA (Silencer Select, Ambion® Life Technologies). A non-targeting siRNA (Ambion® Life Technologies) was used as control. Transfection mixture was added to PC3 cells with P/S-free medium containing 10% FBS (end concentration of siRNA: 50 nM). The medium was changed after 5 h to stop the transfection. For combined knock-down and overexpression analysis, cells were first treated with siRNAs and after 24 h WNT5A overexpression was induced for 24 h.

### RNA isolation, RT, and qPCR

Total RNA was isolated using the HighPure RNA extraction kit (Roche) according to the manufacturer`s protocol. Five-hundred ng RNA were reverse transcribed using Superscript II (Thermo Fisher Scientific) and used for subsequent SYBR green-based quantitative PCR (qPCR) following a standard protocol (Applied Biosystems, Carlsbad, CA, USA). Primer sequences can be found in Supplementary Table 1. PCR conditions were 2 min at 50°C and 10 min at 95°C followed by 40 cycles with 15 s at 95°C and 1 min at 60°C. The melting curve was assessed in the following program: 15 s at 95°C, 1 min at 60°C and 30 s at 95°C. The transcript levels of WNT5A, FZD2, FZD5, FZD6, FZD7, FZD8, ROR2, and RYK (human and mouse) were calculated applying the relative quantification method ΔΔCT [47] and are presented in x-fold increase relative to the house keeping genes GAPDH or β-actin.

### Complementary DNA PCR array

A prostate cancer cDNA Array (III) containing samples of 48 prostatic tissues, including 9 normal prostates, and 39 prostate cancer samples was obtained from Origene (Rockville, USA). Expression of Wnt receptors ROR2, RYK, and FZD5 was assessed and normalized to β-actin using the supplier’s protocol. Prostate cancer samples were sub-divided according to stage grouping (18 stage-II, 19 stage-III, and 2 stage-IV). Also clinical information including GS and TNM staging was provided (for detailed information see www.origene.com).

### Proliferation and apoptosis assays

Cell proliferation was analyzed in PC3 cells using the BrdU Cell proliferation ELISA (Roche), according to the manufacturer`s protocol. For apoptosis studies, the luminescent Caspase Glo^®^ 3/7 Assay (Promega, Mannheim, Germany) measuring the activity of caspases 3 and 7 was used. Assays were performed 48 h after knock-down and/or overexpression treatment. For this purpose, cells were counted after treatment in a 6-well plate and 3.2x10^3^ cells were seeded per well in a 96-well plate. Twenty-four hours after seeding, assays were performed in the 96-well plates.

### Surface plasmon resonance

A Biacore T100 (GE Healthcare, Berlin, Germany) was used for surface plasmon resonance binding studies about WNT5A and its receptors FZD5 and RYK. A Series S sensor chip C1 (GE Healthcare) was used for interaction studies with immobilized WNT5A, while a CM5 sensor chip was used for studies with immobilized FZD5. Therefore, the respective ligands were immobilized onto the sensor chip surfaces by amine coupling at 25°C according to the manufacturers protocol (GE Healthcare). WNT5A (25 μg/ml) was injected for 120 s at a flow rate of 5 μl/min and FZD5 (40 μg/ml) for 840 s at the same flow rate to achieve an immobilization level of about 250 RU in case of WNT5A or 4740 RU in case of FZD5. An activated and afterwards deactivated flow cell served as reference.

The conditions for binding analysis between receptors and ligand were kept constant at 37°C using a flow rate of 30 μl/min. As running buffer and for analyte dilutions, HBS-EP (0.01 M HEPES (pH 7.4), 0.15 M NaCl, 3 mM EDTA, and 0.05% surfactant P20) was used. To avoid the binding of WNT5A to the dextran surface of the reference flow cell, non-specific binding reducer containing 1 mg/ml carboxymethyl dextran (GE Healthcare) was added to the buffer for analyte dilutions. After at least three start-up cycles with running buffer, analytes (recombinant WNT5A, FZD5, RYK; R&D) were injected for a contact time of 240 s. For sequential binding analyses, the first injection of an analyte for 240 s was followed by a 20 s dissociation phase without regeneration prior the second injection of an analyte for 240 s followed by a dissociation time of 1000 s. The respective binding levels were detected 10 s before the end of sample injection relative to the baseline. Regeneration of the chip surface coated with WNT5A or FZD5 was conducted using 4 mM NaOH or 8 M urea for 60 s, respectively. This was followed by a stabilization period of the chip surface for 1000 s using running buffer. The representative sensorgrams were double referenced to the signal of the running buffer and to the reference flow cell. Analysis of binding parameters was performed using the Biacore™ T100 evaluation software 2.03.

### Statistical analysis

Results are presented as the mean ± standard deviation (SD). All *in vitro* experiments were repeated in at least triplicates. Statistical evaluations were performed with GraphPad Prism 7.0 using a one-way analysis of variance (ANOVA) or for single group comparisons using a Student`s *T*-test. Kaplan-Meier estimation was used to determine the probability of overall and disease-specific survival. To compare subgroups, patients were divided at the median expression level into groups with low and high staining scores and the Log-Rank (Mantel-Cox) test was used to compare the probability of overall and disease-specific survival and evaluate the Hazard Ratios. Correlation analysis was performed by Pearson r correlation. *P* values < 0.05 were considered statistically significant.

## SUPPLEMENTARY MATERIALS FIGURES AND TABLES



## Abbreviations

BPH: Benign prostatic hyperplasia
FZD: Frizzled
GS: Gleason Score
LDL: low-density lipoprotein
LRP: LDL receptor related proteins
ROR: Receptor tyrosine kinase-like orphan receptor
RTK: Receptor tyrosine kinase
RYK: Related to receptor tyrosine kinase
TMA: Tissue microarray
SD: standard deviation
WNT5A: Wingless-type MMTV integration site family member 5A
