# Supplementary material for: Role of WNT5A receptors FZD5 and RYK in prostate cancer cells
Source: Oncotarget. 2018 Jun 5;9(43):27293–304. doi: 10.18632/oncotarget.25551 (PMC6007469; doi:10.18632/oncotarget.25551)
Supplement: Supplementary file 1 [file oncotarget-09-27293-s001.pdf]

Role of WNT5A receptors FZD5 and RYK in prostate cancer cells

SUPPLEMENTARY MATERIALS

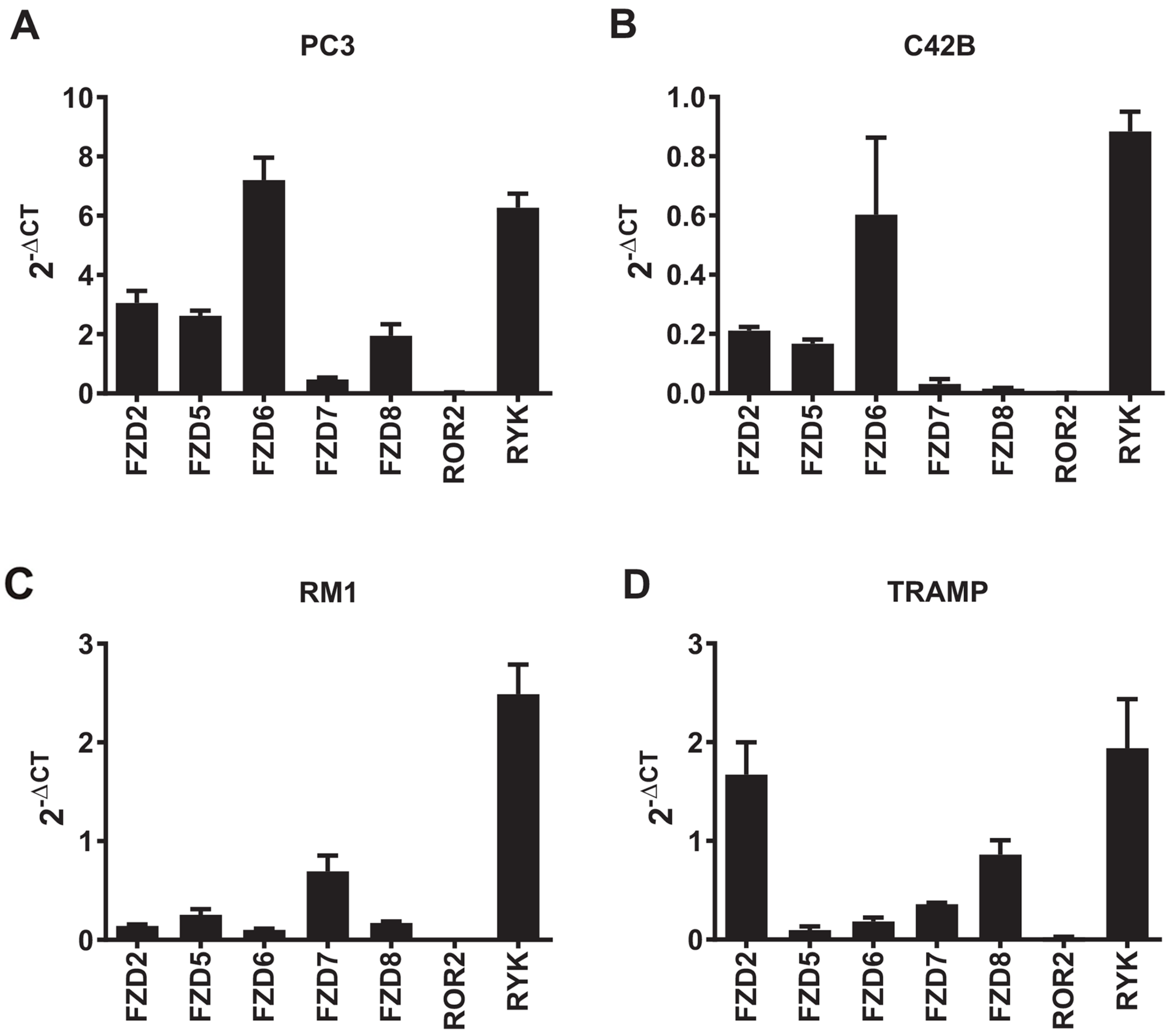

Supplementary Figure 1: Expression of Wnt receptors in human and mouse prostate cancer cell lines. (A-B) Expression of FZD2, FZD5, FZD6, FZD7, FZD8, ROR2, RYK in two different human prostate cancer cell lines PC3 and C4-2B. **(C-D)** Expression of Fzd2, Fzd5, Fzd6, Fzd7, Fzd8, Ror2, Ryk in two different murine prostate cancer cell lines RM1 and TRAMP-C2. The mRNA levels of receptor expression were analyzed by qPCR. Data are shown as $2^{-\Delta CT}$ values as mean ± SD.

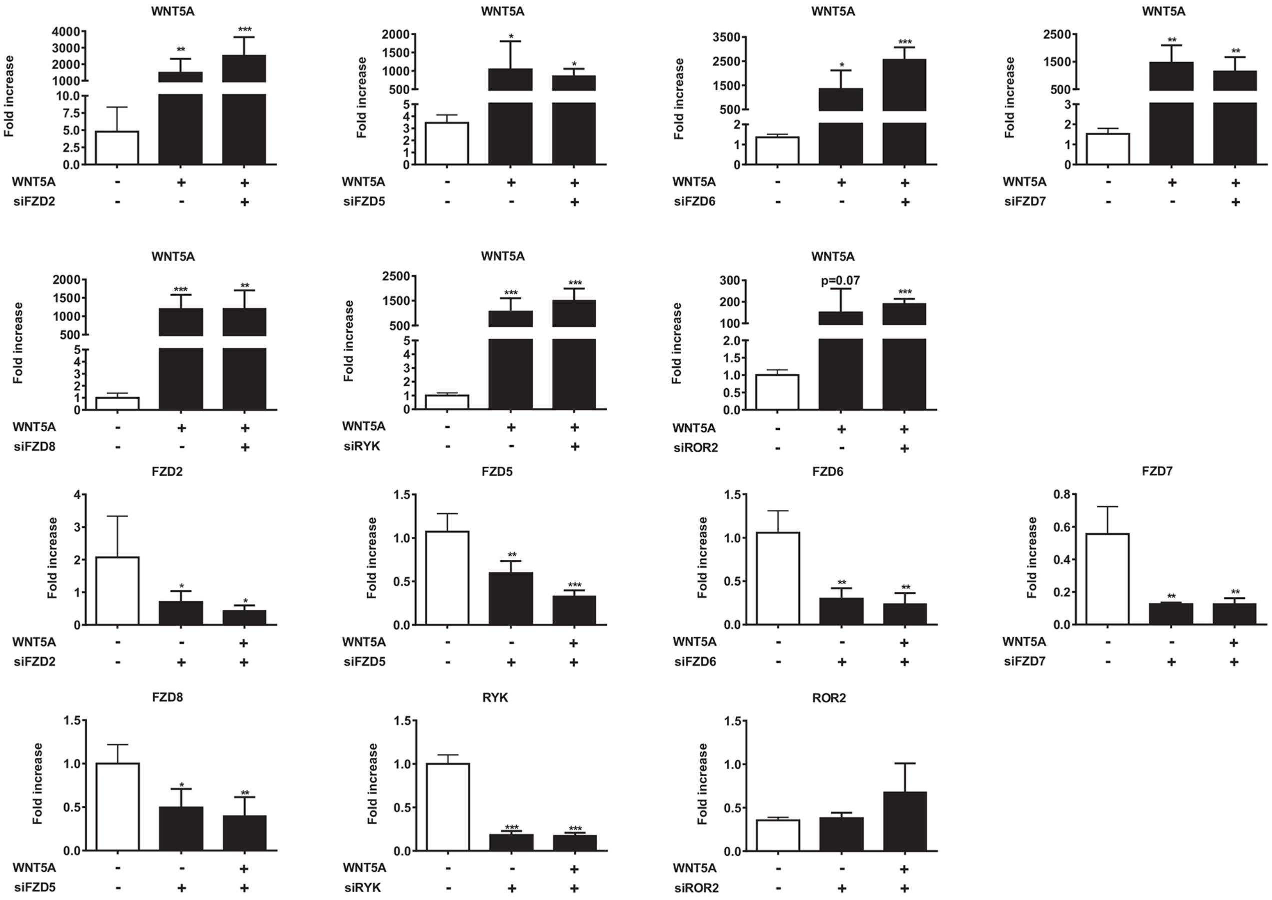

Supplementary Figure 2: Validation of WNT5A overexpression and Wnt receptor knock-downs. PC3 cells were either single treated using siRNAs (siFZD2, siFZD5, siFZD6, siFZD7, siFZD8, siRYK siROR2) or WNT5A plasmid-DNA (pcDNA3.1WNT5A), or treated with a combination of first siRNA (24 h) and afterwards WNT5A plasmid-DNA (24 h). Gene expression of WNT5A and receptors was analyzed using qPCR and normalized to GAPDH. Data are shown as mean ± SD. $^{*}p<0.05$; $^{**}p<0.01$, $^{***}p<0.001$ vs. control.

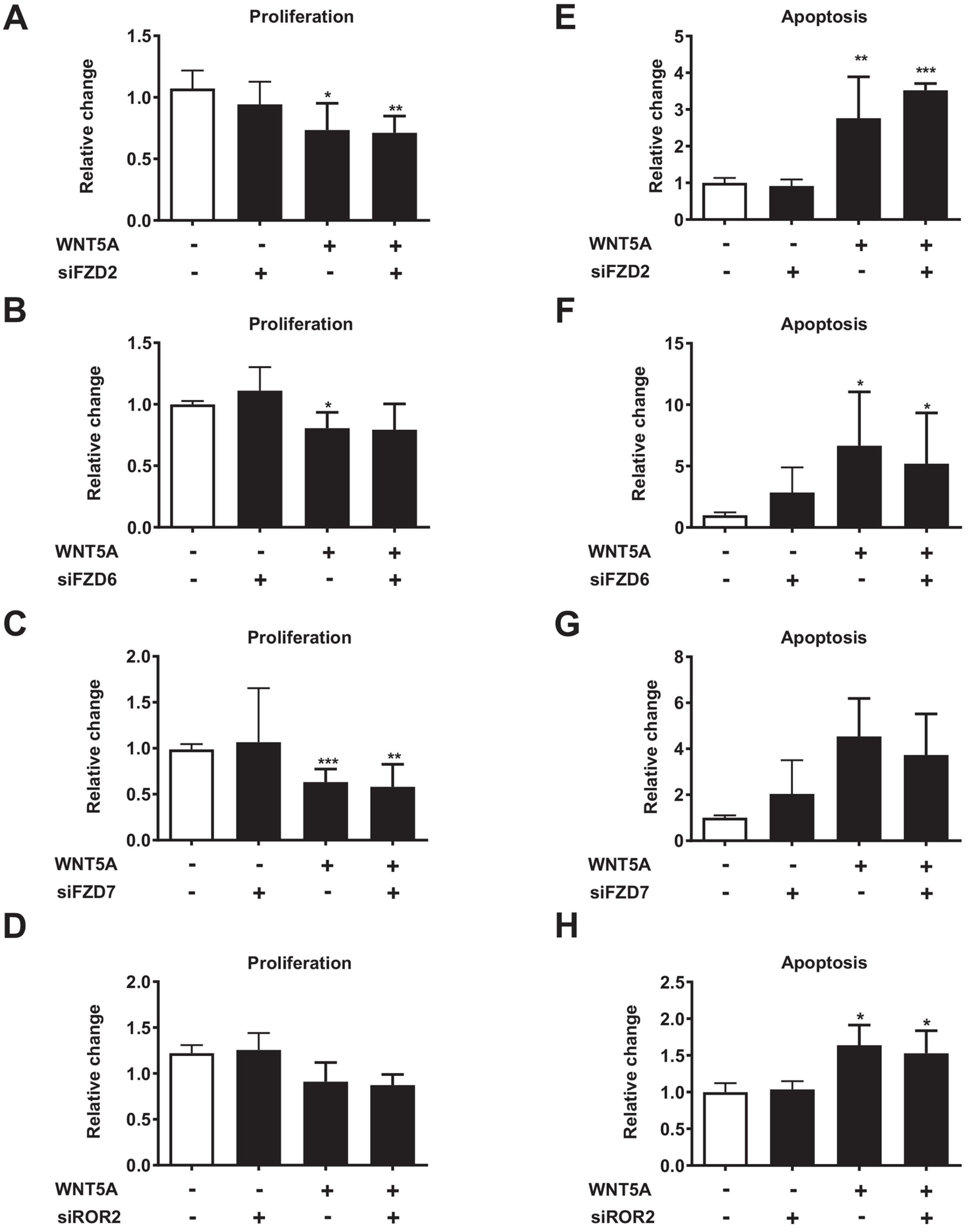

Supplementary Figure 3: Effects of FZD2, FZ6, FZD7 and ROR2 knock-down and/or WNT5A overexpression in PC3 cells. Cells were either single treated using siRNAs or WNT5A plasmid-DNA (pcDNA3.1WNT5A), or treated with a combination of first siRNAs (24 h) and afterwards WNT5A plasmid-DNA (24 h). **(A-D)** Proliferation of control (white bars) and treated (black) PC3 cells. Proliferation was measured using a BrdU proliferation assay after 48 h. **(E-H)** Caspase 3/7 activation of control (white bars) and treated (black) PC3 cells. Apoptosis was measured after 48 h with a Caspase Glo® 3/7 assay. Data are shown as mean ± SD. $^{*}p<0.05$; $^{**}p<0.01$, $^{***}p<0.001$. * vs. control.

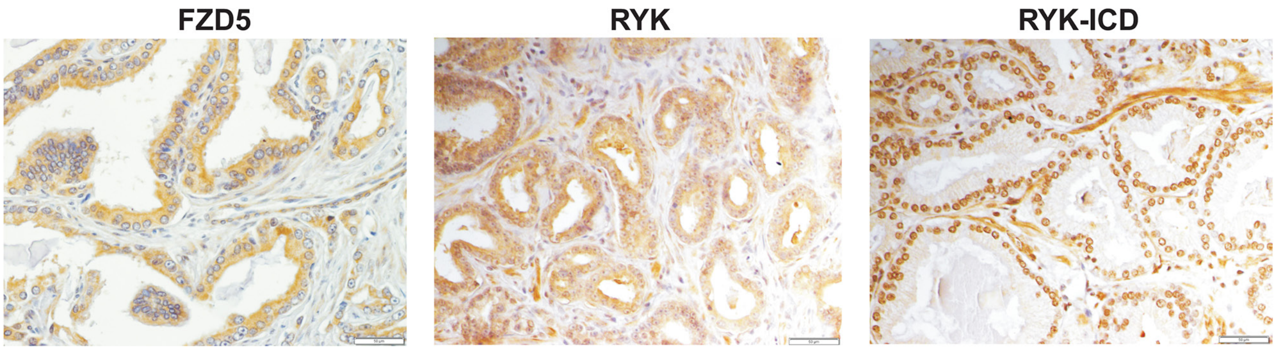

Supplementary Figure 4: Immunohistochemical staining of prostate cancer tissue with FZD5 and RYK. Images show representative staining for FZD5, RYK, and RYK-ICD (nuclear RYK). Scale bar: 50 µm.

Supplementary Table 1: Sequences of human and mouse primer used in this study

Gene	Primer sequences 5'-3	Gene	Primer sequences 5'-3
**Human**		**Mouse**	
FZD2	CCCGACTTCACGGTCTACAT<br>CTGTTGGTGAGGCGAGTGTA	FZD2	GACTCGTTTTGCCCGTCTCT<br>ACCGTGAAGAAAGTGGAAGC
FZD5	AGCTAAAATGGCCAGAGCAA<br>AATTCCCCCTGGGAACTATG	FZD5	GACGCCGAGGTTCTGTGTAT<br>TCCTGGGAGTGTAGGTTTGG
FZD6	TTGTTGGCATCTCTGCTGTC<br>CCATGGATTTGGAAATGACC	FZD6	GAGTCCTGTGAGTTCTTCCTGA<br>ATGATTTGTGGTCGCTCCTG
FZD7	CGACGCTCTTTACCGTTCTC<br>GCCATGCCGAAGAAGTAGAG	FZD7	GCTTCCTAGGTGAGCGTGAC<br>AACCCGACAGGAAGATGATG
FZD8	GACACTTGATGGGCTGAGGT<br>CAAATCTCGGGTTCTGGAAA	FZD8	CTGTTAGCTGGCTTCGTGTC<br>TGTAGAGCACGGTGAAGAGG
ROR2	GGGCAACCTTTCCAACTACA<br>CGTTGCTCACATTGCTCACT	ROR2	CTTCTGCCACTTCGTCTTCC<br>TCCAGCACCTCACATTCATC
RYK	TGATCGGTCTTGATGCAGAA<br>CCAGGTGAAGTGCAGGAAAT	RYK	TTAGCCGTTTTGCACCTTCA<br>TACTGGGTCGTCTGGGTAGA
WNT5A	GGACCACATGCAGTACATCG<br>CCTGCCAAAAACAGAGGTGT	WNT5A	CCAACTGGCAGGACTTTCTC<br>GCATTCCTTGATGCCTGTCT
GAPDH	AGCCACATCGCTCAGACAC<br>GCCCAATACGACCAAATCC	β-Actin	GATCTGGCACCACACCTTCT<br>GGGGTGTTGAAGGTCTCAAA

FZD Frizzled; ROR Receptor tyrosine kinase-like orphan receptor; RYK Related to receptor tyrosine kinase.

Supplementary Table 2: Summary of patient characteristics of the cDNA Array (n=48) and the TMA cohort (n=400)

	**Charactersitics**	**Median (IQR)**
**cDNA Array**	Age at diagnosis (years)	60 (54, 66)
**(n=48)**	**Staging**	**Frequency (%)**
	Healthy	9 (18.75)
	Stage II	18 (37.5)
	Stage III	19 (39.58)
	Stage IV	2 (4.17)
	**Gleason score**	**Frequency (%)**
	Healthy	9 (18.75)
	< 7	2 (4.17)
	= 7	23 (47.92)
	> 7	14 (29.16)
**TMA**	Age at diagnosis (years)	65 (61, 68)
**(n=400)**	Preoperative PSA (ng/ml)	8.6 (5.54, 15.71)
	**TNM staging**	**Frequency (%)**
	pT2	
		154 (38.5)
	pT4	
	pN0	294 (73.5)
	pN1	106 (26.5)
	**Gleason score**	**Frequency (%)**
	< 7	78 (19. 5)
	= 7	75 (18.75)
	> 7	247 (61.75)

IQR, Interquartile range; PSA, prostate-specific antigen; TNM, classification of malignant tumors.
